# TGF-Beta Downregulation of Distinct Chloride Channels in Cystic Fibrosis-Affected Epithelia

**DOI:** 10.1371/journal.pone.0106842

**Published:** 2014-09-30

**Authors:** Hongtao Sun, William T. Harris, Stephanie Kortyka, Kavitha Kotha, Alicia J. Ostmann, Amir Rezayat, Anusha Sridharan, Yan Sanders, Anjaparavanda P. Naren, John P. Clancy

**Affiliations:** 1 Department of Pediatrics, Cincinnati Children's Hospital Medical Center, Cincinnati, Ohio, United States of America; 2 Department of Pediatrics, University of Alabama at Birmingham, Birmingham, Alabama, United States of America; 3 Department of Medicine, University of Alabama at Birmingham, Birmingham, Alabama, United States of America; 4 University of Louisville School of Medicine, Louisville, Kentucky, United States of America; 5 Department of Pediatrics, Nationwide Children's Hospital, Columbus, Ohio, United States of America; 6 Department of Medicine, University of Cincinnati, Cincinnati, Ohio, United States of America; The Ohio State University, United States of America

## Abstract

**Rationale:**

The cystic fibrosis transmembrane conductance regulator (CFTR) and Calcium-activated Chloride Conductance (CaCC) each play critical roles in maintaining normal hydration of epithelial surfaces including the airways and colon. TGF-beta is a genetic modifier of cystic fibrosis (CF), but how it influences the CF phenotype is not understood.

**Objectives:**

We tested the hypothesis that TGF-beta potently downregulates chloride-channel function and expression in two CF-affected epithelia (T84 colonocytes and primary human airway epithelia) compared with proteins known to be regulated by TGF-beta.

**Measurements and Main Results:**

TGF-beta reduced CaCC and CFTR-dependent chloride currents in both epithelia accompanied by reduced levels of TMEM16A and CFTR protein and transcripts. TGF-beta treatment disrupted normal regulation of airway-surface liquid volume in polarized primary human airway epithelia, and reversed F508del CFTR correction produced by VX-809. TGF-beta effects on the expression and activity of TMEM16A, wtCFTR and corrected F508del CFTR were seen at 10-fold lower concentrations relative to TGF-beta effects on e-cadherin (epithelial marker) and vimentin (mesenchymal marker) expression. TGF-beta downregulation of TMEM16A and CFTR expression were partially reversed by Smad3 and p38 MAPK inhibition, respectively.

**Conclusions:**

TGF-beta is sufficient to downregulate two critical chloride transporters in two CF-affected tissues that precedes expression changes of two distinct TGF-beta regulated proteins. Our results provide a plausible mechanism for CF-disease modification by TGF-beta through effects on CaCC.

## Introduction

Regulation of chloride transport is critical to the normal hydration and function of a variety of epithelia, including many of those affected in cystic fibrosis (CF) [1]. Loss of cystic fibrosis transmembrane conductance regulator (CFTR) protein function disrupts chloride transport, with reduced or absent PKA-activated chloride conductance (through CFTR). This loss of CFTR function is frequently associated with an increase in chloride transport through the Calcium activated Chloride Conductance (CaCC) [2]. Research over the past twenty-five years has taught us much about CFTR, which is a chloride and bicarbonate channel that is regulated by regional cyclic adenosine monophosphate (cAMP) and numerous membrane protein interactions [1], [3]–[5]. Much less is known about CaCC which is regulated through surface P_2Y2_ purinergic receptors and ATP [2], [6]. While there is not full agreement regarding the chloride channel identity of CaCC [7], TMEM16A (anoctamin 1) is a recently identified calcium-activated chloride channel that is expressed in many organs affected in CF and may contribute to CaCC [8]. A leading model of CF suggests that function of CaCC can substitute in part for CFTR, providing a redundant chloride-transport pathway to protect organs from lost CFTR activity [9]–[11]. Indeed, *cftr* ^-/-^ mice are largely protected from a spontaneous CF lung phenotype, with upregulated CaCC as a postulated protective mechanism [11]. More recent studies provide evidence that changes in CaCC activity can influence the CF phenotype in human subjects. For example, CaCC downregulated by estradiol may contribute to accelerated decline in lung function in post-pubescent CF females [12]. In addition, downregulation of CaCC during respiratory viral infections may disrupt mucociliary clearance in the CF airway and contribute to pulmonary exacerbations [13], [14]. These data support the notion that changes in CaCC activity are directly relevant to the CF phenotype.

TGF-beta is an established genetic modifier in CF [15]–[18], but it is unclear how it influences CF disease. It is a pleotropic signaling molecule that is often upregulated under conditions of tissue injury and repair, and pathologic upregulation of TGF-beta is a known contributor to fibrogenic diseases [19], [20]. TGF-beta can drive changes in the expression of epithelial and mesenchymal genes in differentiated epithelia [19], [21] that result in a motile fibroblastoid phenotype [20]. This may be an important aspect of tissue healing and regeneration, but dysregulation can lead to tumorigenesis and tissue fibrosis [19]–[24].

Previous studies have provided evidence that TGF-beta downregulates CFTR expression in polarized T84 human colonocytes (T84 cells) and in primary human-airway epithelial cells (HAECs) through p38 MAPK- and Smad-2/3-dependent mechanisms, respectively [25], [26]. How this could influence the CF phenotype, where CFTR function is low or absent, is not clear. In contrast, relationships between TGF-beta, CaCC, and TMEM16A have not been examined, nor have the simultaneous effects of TGF-beta on ion transporters and other TGF-beta-regulated processes in CF-affected epithelia.

In this report we examined TGF-beta effects on these chloride transport pathways in T84 cells and HAECs, which are two well-established model systems to study CaCC and CFTR. We also examined dose/response relationships between TGF-beta and TMEM16A, CFTR and proteins known to be regulated by TGF-beta (e-cadherin, vimentin, alpha smooth muscle actin). TGF-beta was sufficient to suppress calcium-activated and cAMP-activated chloride transport, to inhibit the expression of TMEM16A and CFTR, and to reverse F508del CFTR correction produced by VX-809 treatment (a known corrector of F508del CFTR trafficking to the plasma membrane that is currently in clinical trials) [27], [28]. These effects were observed at TGF-beta concentrations below those necessary to affect the expression of e-cadherin and vimentin. The TGF-beta effects on TMEM16A and CFTR expression were partially reversed by Smad3 and p38 MAPK inhibition, respectively. These data support the hypothesis that TGF-beta can modify the CF phenotype through broad downregulation of chloride transporters in CF-affected epithelia via distinct signaling pathways.

## Methods

### Cell culture

T84 cells were obtained from the ATCC (Manassas, VA), grown in T75 flasks (Costar, St. Louis, MO), seeded onto collagen-coated Transwell filters (6.5 mm; Corning, Inc., Corning, NY), and maintained in DMEM medium containing 10% FBS (Life Technologies, Grand Island, NY). Resistance was monitored using EVOM^2^ (World Precision Instruments, Sarasota, FL), and cells were studied when resistance was at 500–1000 Ω.cm^2^. Cells were serum starved (0.1% FBS) for 24 h prior to TGF-beta exposure. HAECs obtained from the University of North Carolina Airway Cell Core (under the direction of Dr. Scott Randell) were isolated from donor or recipient lungs that were cryopreserved at passage 1 and cultured as previously described [29]. Briefly, HAECs were cultured in Bronchial Epithelial Growth Medium (BEGM; Lonza, Hopkinton, MA) on Purecol-coated tissue-culture dishes (Advanced Biomatrix, San Diego, CA) until 80–90% confluent, then passaged and plated onto Transwell-Clear permeable supports (0.4 µm pore size; Corning, Inc.) coated with type IV collagen (Sigma-Aldrich, St. Louis, MO). To correct F508del CFTR trafficking, F508del/F508del HAECs were treated with VX-809 (3 µM; Selleck Chemicals, Houston, TX) for 48 h prior to study [27], [28].

### Ion transport

Short-circuit current (I_sc_) and resistance were measured using Acquire and Analyze 2.3 software (San Diego, CA) as previously described [30]. In T84 cells, CFTR was activated with forskolin/IBMX (10/100 µM), and the CFTR current was augmented by basolateral stimulation of potassium channels with carbachol (100 µM, causing cell hyperpolarization to drive anion exit). This is a well-established method to maximize the detection of CFTR in the luminal membrane of colonic epithelia [31]–[33]. In HAECs, CFTR was activated with forskolin/IBMX (10/100 µM) and then potentiated with genistein (50 µM). Calcium-activated chloride currents were stimulated by ionomycin (2 µM) followed by basolateral carbachol (100 µM) in both T84 cells and HAECs. CFTR_inh172_ [34] and tannic acid [7] were used to block CFTR and CaCC currents, respectively. For permeabilization studies, 50 µg/ml nystatin was added to the basolateral membrane as previously described [35].

### Airway Surface Liquid (ASL) measurements

ASL volume was measured by the method published by Harvey and colleagues with minor modifications [36].

### Immunoblot

Immunoblot was performed using methods similar to those previously described [30]. Primary antibodies (mouse anti-CFTR 570 1∶4000 or rabbit anti-TMEM16A 1∶1000; rabbit anti-e-cadherin 1∶1000 or mouse anti-vimentin 1∶1000; mouse anti-alpha smooth muscle actin) were added to the blots and incubated overnight at 4°C, then horseradish peroxidase-conjugated secondary antibodies (goat anti-mouse or goat anti-rabbit 1∶200,000) were applied in PBS. Immunoreactivity was detected by chemiluminescence (SuperSignal West Fretmo, Thermo Scientific, Rockford, IL) and quantitated using NIH Image software (Bethesda, MD).

### Real time RT-PCR

TaqMan One Step RT-PCR (Applied Biosystems Life Technologies, Grand Island, NY) was used to quantify CFTR and TMEM16A mRNA transcripts using “*Assays on Demand*” Gene Expression Products (Applied Biosystems Life Technologies). RT-PCR was performed using methods similar to those previously described [30].

### cAMP and calcium measurements

See Online Data (Figure S2).

### Immunoflourscence

See Online Data (Figure S4).

### Statistical analyses

Paired and unpaired t-tests or ANOVA (as appropriate) were used to compare continuous data, including I_sc_, densitometry, and PCR, using Sigmastat software (San Jose, CA). An alpha value of 0.05 was used to determine the statistical significance of obtained *P* values.

## Results

### TGF-beta downregulates calcium and cAMP- stimulated chloride currents in T84 cells and HAECs

**Figure 1:**
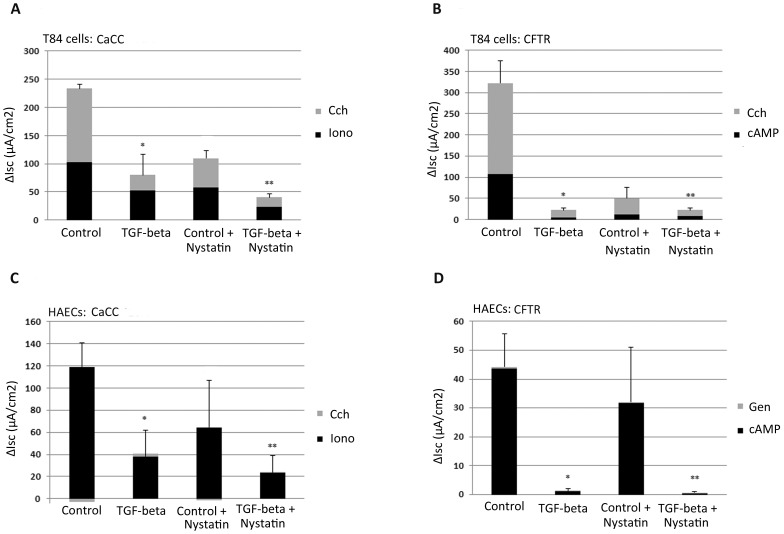
Downregulation of both CaCC and CFTR currents in T84 cells and HAECs by TGF-beta. T84 (**A and B**) and HAECs (**C and D**) were treated with TGF-beta (10 ng/ml) for 48 h prior to I_sc_ measurement and studied as described in the Methods and as shown in Figure S1. T84 cells without nystatin were studied with symmetric apical and basolateral buffers. All other T84 and HAEC studies were completed with a basolateral-to-apical chloride secretory gradient [27]. Briefly, to activate CaCC-dependent chloride transport in T84 monolayers (**A**), cells were stimulated with ionomycin (Iono; 2 µM) to raise calcium and carbachol (CCh; 100 µM, basolateral) to activate basolateral potassium channels and drive apical chloride exit. Similar stimuli were used in HAECs (**C**) but carbachol had little effect on stimulated Isc. To activate CFTR-dependent chloride transport in both cell types (**B and D**), cells were stimulated with forskolin/IBMX (10 µM/100 µM) to raise cAMP. In T84 cells (**B**), carbachol (100 µM, basolateral) was used to increase apical chloride transport similar to A. In HAECs (**D**), genistein (50 µM, apical) was used to potentiate CFTR. The right two bars in each panel represent studies with a chloride secretory gradient following permeabilization of the basolateral membrane with nystatin (50 µg/ml). (**A**) *P = 0.001compared with control; **P = 0.007 compared with nystatin control. (**B**) *P = 0.003 compared with control; **P = 0.015 compared with nystatin control. (**C**) *P = 0.041 compared with control; **P = 0.016 compared with nystatin control. (**D**) *P = 0.006 compared with control; **P = 0.014 compared with nystatin control.

Previous reports indicate that TGF-beta treatment downregulates CFTR expression and activity in T84 cells and HAECs [25], [26], [37]. We tested the hypothesis that TGF-beta would downregulate the expression and function of both CaCC and CFTR using published TGF-beta exposure. Representative control experiments (i.e., no TGF-beta exposure) are shown in Figure S1A-D, and summarized results of TGF-beta effects on chloride channel function are shown in Figure 1A–D. TGF-beta treatment (10 ng/ml) of T84 cells for 48 h dramatically reduced currents through CaCC following ionomycin + basolateral carbachol (2 µM and 100 µM, respectively, *P*<0.001), and CFTR currents following forskolin/IBMX + basolateral carbachol (10/100 µM and 100 µM, respectively, *P* = 0.003) relative to control conditions (Figure 1A and B). The inhibitory effects of TGF-beta on chloride conductance persisted following basolateral membrane permeabilization with nystatin, confirming that TGF-beta inhibited both apical plasma-membrane chloride channels. Similar experiments conducted in HAECs are shown in Figure 1C and D. TGF-beta treatment for 48 h inhibited both CaCC activity following ionomycin + basolateral carbachol (*P* = 0.041) and CFTR currents following stimulation with forskolin/IBMX + apical genistein (*P* = 0.006) relative to control conditions. These inhibitory effects also persisted in HAECs following basolateral membrane permeabilization with nystatin. The inhibitory effects of TGF-beta were less pronounced for CaCC compared with those for CFTR-dependent currents. CaCC-dependent currents were reduced 66% and 71.8% in T84 cells and HAECs, respectively. In contrast, CFTR activity following TGF-beta treatment was reduced 93.2% and 98% in the two cell types. While not a direct goal of our studies, we also observed that amiloride-sensitive currents were potently reduced by TGF-beta treatment of HAECs (from −11.3±2.3 to −0.875±0.25 µA/cm^2^, *P*<0.001). Since ENaC expression is typically low/absent in T84 cells, we focused the remainder of our studies on CaCC and CFTR across the two cell types.

**Figure 2:**
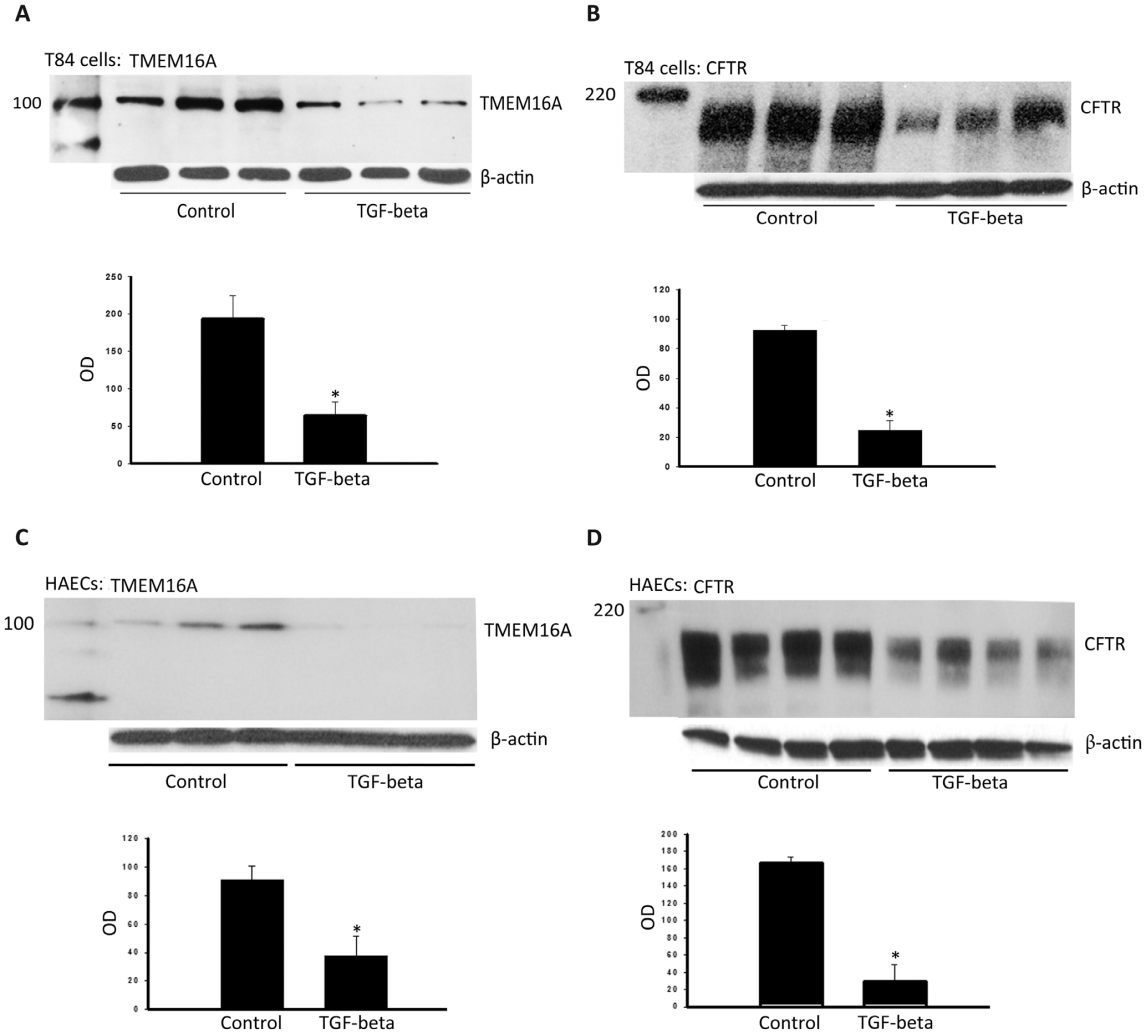
TGF-beta effects on TMEM16A and CFTR protein levels in T84 cells and HAECs. Lysates of T84 cells (**A and B**) or HAECs (**C and D**) were prepared and subjected to PAGE and immunoblot with either anti-TMEM16A or anti-CFTR antibody. For each cell type, the upper gel panel shows TMEM16A (**A and C**) or CFTR (**B and** **D**) detection from three replicate samples (with or without 10 ng/ml TGF-beta exposure). The lower panels are summary densitometry data. **T84 cells:** *P = 0.01 for TMEM16A; *P = 0.01 for CFTR. **HAECs:** *P = 0.05 for TMEM16A; *P = 0.01 for CFTR.

Complementary immunoblots of TMEM16A and CFTR confirmed that expression of both chloride channels was reduced in both T84 cells and HAECs after 48 h of TGF-beta treatment (Figure 2A and B - T84 cells, Figure 2C and D - HAECs). The effects of TGF-beta on protein levels of both channels (by densitometry) generally corresponded with the effects observed on I_sc_. TGF-beta reduced TMEM16A expression 67% and 59% in T84 cells and HAECs, respectively, compared with reductions of CFTR detection by 74% and 83% in the two cell types.

**Figure 3:**
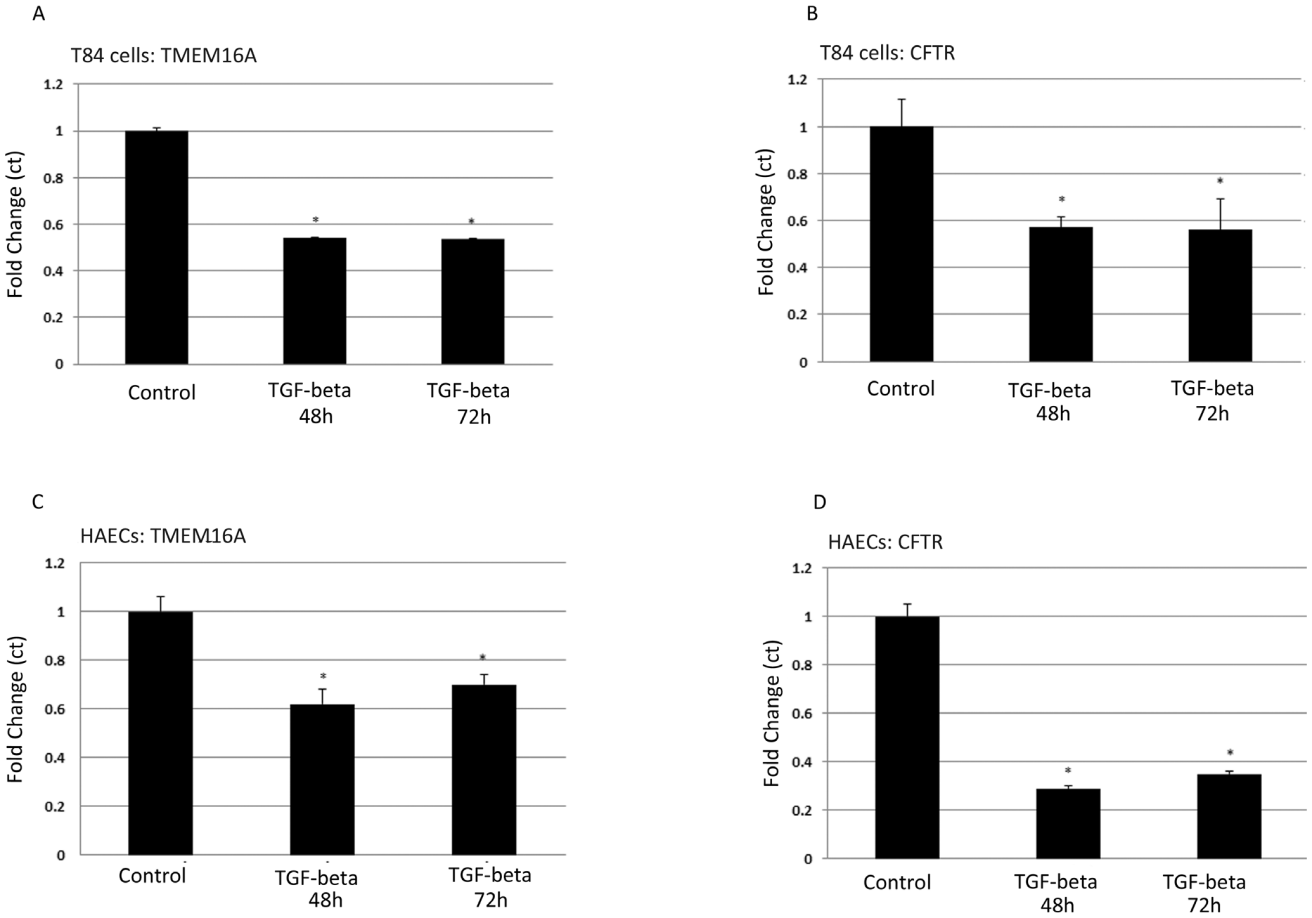
TGF-beta effects on TMEM16A and CFTR mRNA levels in T84 cells and HAECs. Relative transcript levels of TMEM16A from T84 cells (**A**) and HAECs (**C**) under both control conditions and exposure to 10 ng/ml of TGF-beta. *P = 0.001 and 0.001 for T84 cells at 48 and 72 h; *P = 0.024 and 0.026 for HAECs at 48 and 72 h. Relative transcript levels of CFTR from T84 cells (**B**) or HAECs (**D**) under both control conditions and exposure to 10 ng/ml of TGF-beta. *P = 0.045 and 0.019 for T84 cells at 48 and 72 h; *P = 0.026 and 0.035 for HAECs at 48 and 72 h.

In addition to downregulated chloride-channel function and protein levels (Figures 1 and 2), TGF-beta also reduced TMEM16A and CFTR transcription in both cell types (Figure 3A–D). Treatment with TGF-beta produced maximal reductions of TMEM16A and CFTR transcript levels within 48 h relative to untreated cells, with no further reduction following 72 h of exposure. The effects of TGF-beta on TMEM16A and CFTR transcripts were less pronounced relative to effects on protein levels and function of both chloride channels. For T84 cells, transcript levels for TMEM16A and CFTR were reduced approximately 40–50% relative to untreated controls. For HAECs, CFTR transcripts were more sensitive to TGF-beta exposure (∼70% reduction observed within 48 h) than TMEM16A transcripts (reduced ∼40%) relative to control conditions. These effects of TGF-beta on chloride transport and channel expression are summarized in Table 1.

**Table 1:** Summary of TGF-beta inhibition of CaCC and CFTR in T84 cells and HAECs.

	T84 cells			HAECs		
**Channel**	I_sc_	Protein	RNA	I_sc_	Protein	RNA
**CaCC** *	61±11.88	67.67±4.78	46.1±0.26	67.25±7.25	61.±11.04	38±0.6
**CFTR**	93.8±2.13	74±6.48	45.67±10.33	97±1.4	83.3±10.14	71±14

Values are % reduction (±SD) produced by TGF-beta exposure (10 ng/ml, 48 h).

*TMEM16A protein and RNA levels are provided for comparative purposes.

### TGF-beta disrupts ASL regulation in polarized HAECs

**Figure 4:**
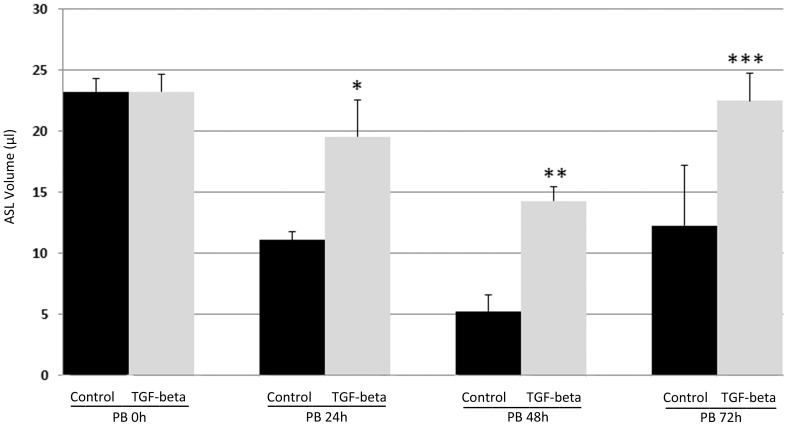
TGF-beta disruption of airway surface liquid (ASL) regulation in HAECs. Polarized non-CF HAECs had apical fluid removed, and the apical surface was then bolused with 20 µl of media. Cells were treated with vehicle (control) or TGF-beta (10 ng/ml), and the ASL volume was measured at 0, 24, 48, and 72 h post bolus (PB). TGF-beta or vehicle (diluted in water) was added to the to the apical bolus media at time  =  0, and to the basolateral media at time  =  0, 24 and 48 hours. N = 6. *P = 0.02 (control vs TGF-beta); **P = 0.00043 (control vs TGF-beta); ***P = 0.07 (control vs TGF-beta).

The ASL volume of polarized HAECs has been shown to be auto regulated by several ion transporters, including ENaC, CFTR, and CaCC [10], [38]. To examine the effect of TGF-beta on this critical airway epithelial function, polarized HAECs (non-CF) cultured ex-vivo for six weeks were treated with TGF-beta (10 ng/ml) or vehicle for 72 h following a 20 µl apical bolus with media (Figure 4). Under control conditions, the ASL volume dropped to ∼12 µl within 72 h. In the presence of TGF-beta, autoregulation of the ASL volume was reduced over 72 h of treatment compared with the control conditions. In parallel, TGF-beta exposure (10 ng/ml) significantly increased monolayer resistance in both cell types over 72 h of exposure [T84 cells control  = 556.6Ω·cm^2^ (±180 SD), T84 cells + TGF-beta  =  1,729.7Ω·cm^2^ (±292.7; *P*<0.001); HAECs control  =  759.8Ω·cm^2^ (±145.4), HAECs + TGF-beta  =  1,793.5Ω·cm^2^ (±349.8; *P*<0.001)]. These results are consistent with the TGF-beta-downregulation of ion-transporter expression and function demonstrated in Figures 1–4.

### TGF-beta does not downregulate cAMP or calcium production in either T84 cells or HAECs

We next tested whether stimulated cAMP or calcium levels were reduced by TGF-beta treatment, thus potentially contributing to the greater functional inhibition of CFTR and CaCC (Figure 1A–D) relative to mRNA and protein levels (Figures 2A–D and 3A–D, respectively). T84 cells and HAECs were treated with TGF-beta (10 ng/ml) or vehicle for 48 h and then stimulated with either ionomycin (2 µM) or forskolin/IBMX (10 µM/100 µM) for 5 min (Figure S2A–D). TGF-beta treatment did not reduce calcium or cAMP generation in either cell type, confirming that the inhibitory effects of TGF-beta on either chloride channel were independent of calcium and cAMP production.

### TGF-beta inhibits F508del CFTR correction by VX-809

**Figure 5:**
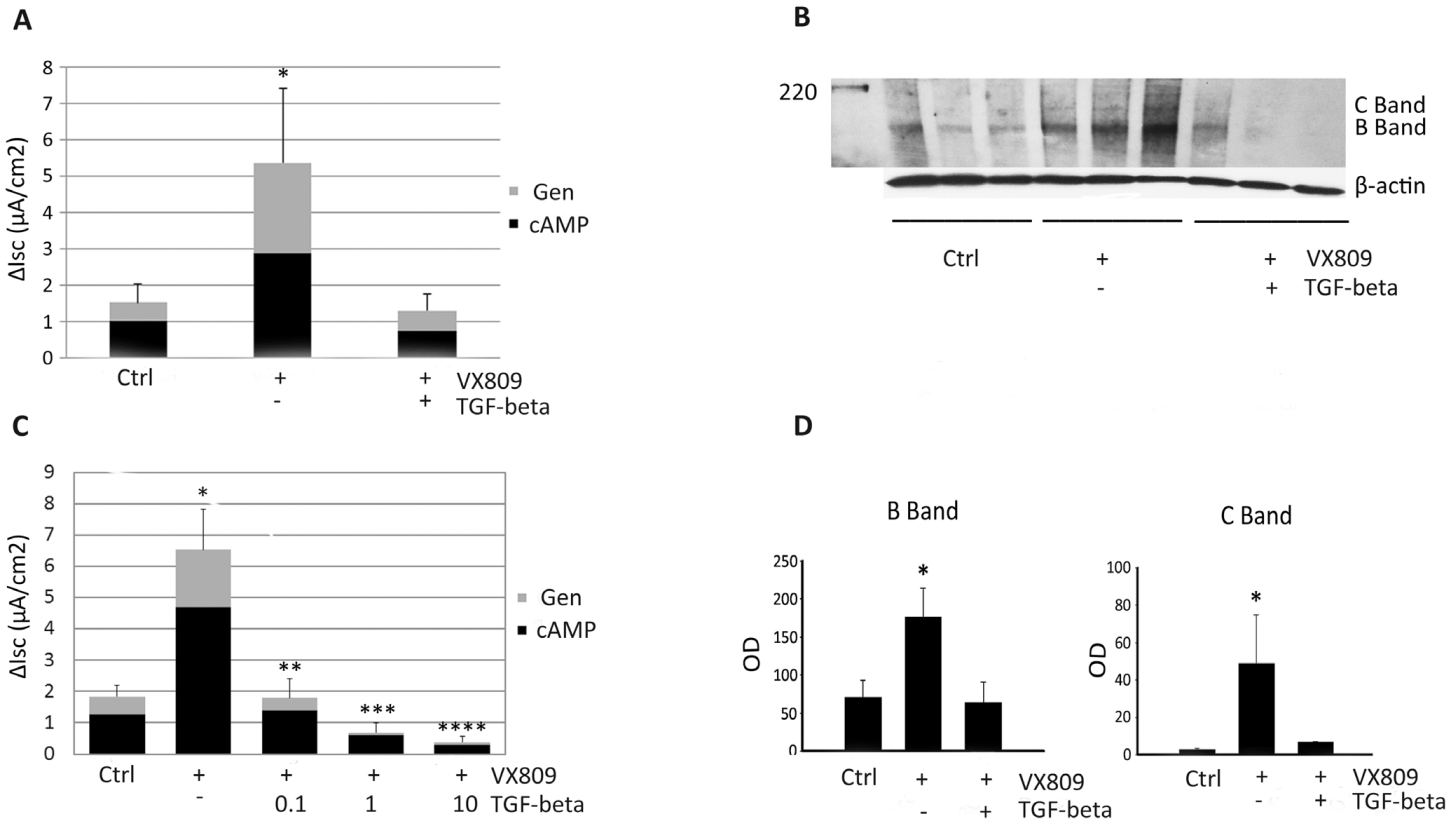
TGF-beta downregulation of VX-809-corrected F508del CFTR in HAECs. F508del CFTR homozygous HAECs were grown at air-liquid interface culture for 6 weeks and treated with TGF-beta (10 ng/ml) and/or VX-809 (3 µM) for 48 h prior to I_sc_ measurement. (**A**) Stimulated I_sc_ under control (left), VX-809 alone (middle), or VX-809 + TGF-beta (right) treatment conditions. *P<0.035, VX-809 compared with either control or VX-809 + TGF-beta. (**B**) Lysates of F508del HAECs from each filter were prepared and subjected to PAGE and immunoblot as described in Methods for detection of B Band and C Band under control, VX-809 alone, or VX-809 + TGF-beta treatment conditions. (**C**) All tested doses of TGF-beta downregulated VX-809-corrected F508del CFTR. *P<0.00017, VX-809 compared with untreated control; **P<0.0001, 0.1 ng TGF-beta + VX-809 compared with VX-809 alone. ***P<0.0001, 1 ng TGF-beta + VX-809 compared with VX-809 alone. ****P<0.0001, 10 ng TGF-beta + VX-809 compared with VX-809 alone. (**D**) Summary densitometry data for detection of F508del CFTR B Band (left) and C Band (right) with and without TGF-beta (10 ng/ml, similar to 5B). *P≤0.047, B Band VX-809 compared with either control or VX-809 + TGF-beta. *P≤0.05, C Band VX-809 compared with either control or VX-809 + TGF-beta.

Recent studies indicate that VX-809 partially restores F508del CFTR trafficking in both HAECs and human subjects [27], [28]. Treatment of primary F508del/F508del HAECs with VX-809 (3 µM, 48 h) effectively increased F508del CFTR activity and levels of F508del CFTR B Band and C Band (Figure 5B and D). In agreement with a previous report [26], these effects were completely reversed by co-treatment with TGF-beta (10 ng/ml, 48 h) (Figure 5A). Dose-response studies (Figure 5C) demonstrated that VX-809-corrected F508del CFTR was exquisitely sensitive to TGF-beta exposure, with complete abrogation of F508del CFTR activity following 0.1 ng/ml exposure (48 h, *P*<0.0001).

### Downregulation of TMEM16A and CFTR by TGF-beta precedes expression changes in proteins regulated by TGF-beta

**Figure 6:**
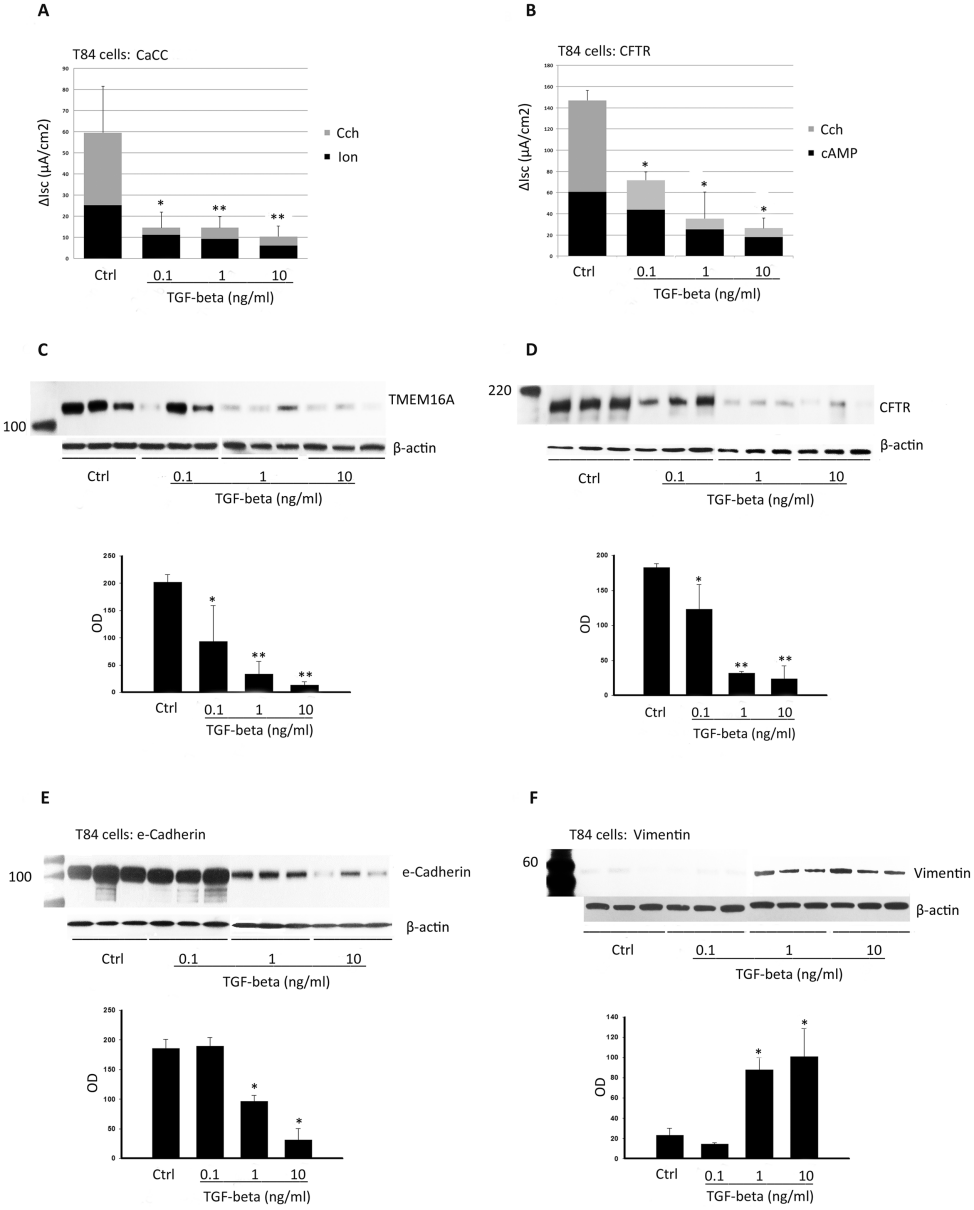
Dose-response effects of TGF-beta on TMEM16A, CFTR, e-cadherin, and vimentin in T84 cells. T84 cells were treated with TGF-beta (vehicle control, 0.1, 1.0, and10 ng/ml) for 48 h, followed by Ussing chamber and immunoblot as described in Methods. (**A and B**) CaCC (*P<0.003, **P<0.01) and CFTR (*P<0.0001) currents were reduced by TGF-beta treatment at every dose used. (**C and D**) TMEM16A (*P<0.01, **P<0.005) and CFTR (*P<0.05, **P<0.0001) expression was suppressed by TGF-beta treatment at every dose used. E-cadherin expression (**E**) was decreased (*P<0.011) and vimentin expression (**F**) was increased (*P<0.05) with 1 ng/ml or greater TGF-beta treatment.

**Figure 7:**
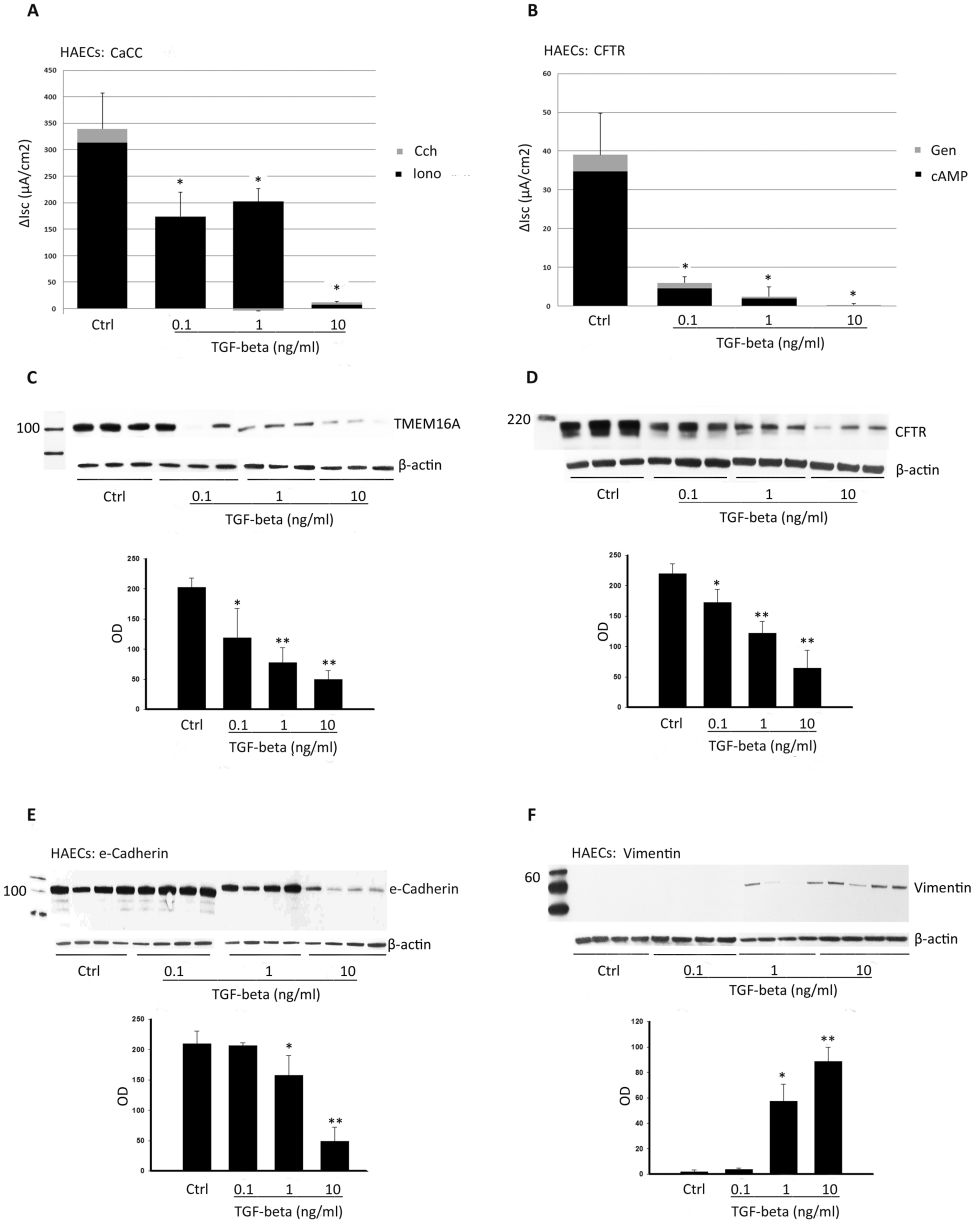
Dose-response effects of TGF-beta on TMEM16A, CFTR, e-cadherin, and vimentin in HAECs. HAECs were treated with TGF-beta (vehicle control, 0.1, 1.0, and10 ng/ml) for 48 h, followed by Using chamber and immunoblot as described in Methods. (**A and B**) CaCC (*P<0.0016) and CFTR (*P<0.0001) currents were reduced by TGF-beta treatment at every dose used. (**C and D**) TMEM16A (*P<0.045, **P<0.02) and CFTR (*P<0.04, **P<0.006) expression was suppressed by TGF-beta treatment at every dose used. E-cadherin expression (**E**) was decreased (*P<0.006, **P<0.0001) and vimentin expression (**F**) was increased (*P = 0.001, **P<0.0003) with 1 ng/ml or higher TGF-beta treatment.

TGF-beta is known to influence the expression of several epithelial and mesenchymal proteins [19]–[21], and in our hands, corrected F508del CFTR was highly sensitive to TGF-beta exposure. This raised the question of whether TGF-beta effects on chloride transporter expression and function could precede it's effects on other proteins established to be regulated by TGF-beta. To address this question, we examined dose/response relationships between TGF-beta exposure and TMEM16A and CFTR expression relative to an epithelial protein marker (e-cadherin) and mesenchymal protein markers (vimentin and alpha smooth muscle actin). All of these markers are well known to be regulated by TGF-beta [19]–[21]. As shown in Figure 6A-D, reductions in CaCC and CFTR expression and function in T84 cells were observed at TGF-beta doses as low as 0.10 ng/ml (*P*<0.003), while changes in e-cadherin and vimentin expression were undetectable until 1 ng/ml TGF-beta exposure (Figure 6E and F). Similar findings were observed in HAECs, with statistically significant reductions in TMEM16A and CFTR expression at 0.1 ng/ml of TGF-beta (Figure 7A–D, *P*<0.05), while changes in e-cadherin and vimentin expression were not observed until 1 ng/ml TGF-beta exposure (Figure 7E and F). Expression of an additional TGF-beta-regulated protein (alpha smooth muscle actin) was not detected in either epithelial cell type, with or without TGF-beta exposure (data not shown). The effect of TGF-beta on additional airway epithelial markers and morphology by immunofluorescence is shown in Figure S4A–B. TGF-beta treatment (10 ng/ml, 48 h) disrupted normal e-cadherin staining, but had little effect on MUC5AC or acetyl tubulin detection.

### Reversal of TGF-beta-inhibited TMEM16A and CFTR expression by pSmad3 and p38 MAPK blockers

**Figure 8:**
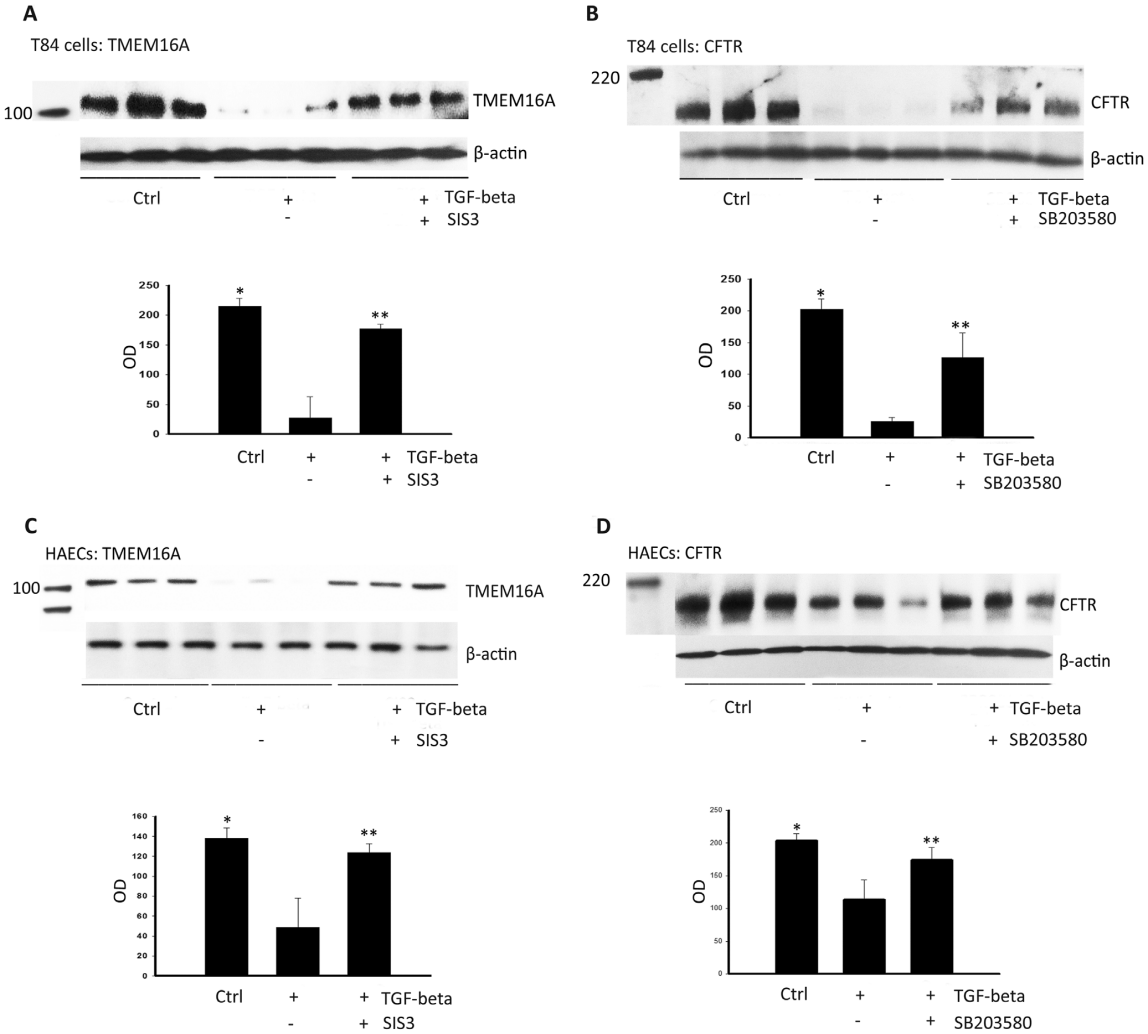
Rescue of TGF-beta-downregulated TMEM16A expression by a Smad3 inhibitor (SIS3) and CFTR expression by a p38 MAPK inhibitor (SB203580). T84 cells and HAECs were treated with either TGF-beta and SIS3 (5 µM, A and C) or TGF-beta and SB203580 (10 µM, B and D) for 48 h prior to lysis and immunoblot for TMEM16A (A and C) or CFTR (B and D). For each cell type, the upper gel panels show TMEM16A (A and C) or CFTR (B and D) detection from three replicate samples (with TGF-beta and either SIS3 or SB203580). SIS3 increased TMEM16A expression and SB203850 increased CFTR expression from TGF-beta-treated T84 cells and HAECs. The lower panels are summary densitometry data. T84 cells: *P<0.0002 for TMEM16A and CFTR control vs TGF-beta. **P<0.0007 for CFTR and TMEM16A + SB203580 and TGF-beta vs TGF-beta alone. HAECs: *P<0.035 for TMEM16A and CFTR control vs TGF-beta. **P<0.05 for CFTR and TMEM16A + SB203580 and TGF-beta vs TGF-beta alone.

To examine whether common or separate TGF-beta cell-signaling pathways were responsible for inhibition of TMEM16A and CFTR expression, we treated cells with selective inhibitors of Smad3 and p38 MAPK during TGF-beta exposure (10 ng/ml). TGF-beta exposure upregulated the expression of both pSmad3 and phosphor p38 in both cell types (Figure S3A–D). Smad3 inhibition (SIS3, 5 µM) restored TGF-beta-downregulated TMEM16A expression in both T84 cells and HAECs (Figure 8A and C) (*P*<0.0002), while inhibition of p38 MAPK (SB203580, 10 µM) restored TGF-beta-downregulated CFTR expression in both cell types (Figure 8B and D) (*P*<0.035). Control experiments demonstrated that p38 MAPK inhibition had no effect on TMEM16A expression, and Smad3 inhibition had no effect on CFTR expression in either cell type following TGF-beta exposure (Figure S5A–D).

## Discussion

In the current study, we examined intersections between TGF-beta exposure and calcium- and cAMP-activated chloride currents, TMEM16A and CFTR expression, and dose/response relationships between TGF-beta-regulated proteins in two CF-affected cell types. We hypothesized that TGF-beta may have broad and potent effects on chloride transport in both airway and colonic epithelia. Our findings demonstrate clearly that TGF-beta is a strong negative regulator of both CaCC and CFTR, which are the two key epithelial chloride transporters relevant to CF pathogenesis. The two pathways were regulated by separate transcription factors (pSmad2/3 and p38 MAPK), and their sensitivity to TGF-beta was greater than that of common markers reflective of TGF-beta signaling (e-cadherin and vimentin). The results confirm reports by Howe and colleagues [25] describing downregulation of CFTR by TGF-beta in T84 cells. Similar findings in the porcine vas deferens have been reported by Pierucci-Alves and colleagues [39], and both of these studies identified p38 MAPK as a contributor to TGF-beta inhibition of CFTR. Our data also confirm recent reports in HAECs [26], [37], including downregulation of wtCFTR and F50del CFTR currents and expression produced by VX-809.

Our results extend prior work in this area in at least two critical ways, demonstrating that i) TGF-beta inhibitory effects extend to CaCC and TMEM16A in both colonocytes (T84 cells) and HAECs, and ii) TGF-beta inhibition of CaCC, TMEM16A and CFTR occurred at low doses relative to established effects of TGF-beta on other epithelial proteins. While TGF-beta treatment upregulated expression of a mesenchymal marker (vimentin) and downregulated an epithelial marker (e-cadherin), changes of complete epithelial:mesenchymal transition were not seen over the time course and dosing used in our experiments. We did not observe induction of alpha smooth muscle actin expression in either cell type, and HAECs continued to express MUC5AC and the cilia marker acetyl tubulin. The effects on CaCC, TMEM16A and CFTR were observed at 10-fold lower doses of TGF-beta than necessary to induce changes in other proteins regulated by TGF-beta (Figures 6 and 7). These results indicate that TMEM16A and CFTR are highly sensitive to TGF-beta exposure. In addition, our data support the notion that these chloride channels may be sensitive to tissue-specific conditions of increased TGF-beta activity. Although our studies were not designed to clarify the relative contribution of TMEM16A to CaCC, they do demonstrate the similar sensitivity of both TMEM16A and CaCC to TGF-beta downregulation in both T84 cells and HAECs.

Previously, it has been reported that TGF-beta levels in the bronchoalveolar lavage (BAL) fluid of CF patients were nearly three-fold higher than that observed in non-CF patients (135±15 pg/ml vs 57±10 pg/ml, *P*<0.01) [40], with levels further increased in CF patients with diminished lung function [increased compared to CF patients with normal lung function (205.9±20.5 pg/ml vs 106.4±24.0 pg/ml, *P*<0.01)] [41]. Since the epithelial lining fluid is estimated to be diluted over 100-fold in BAL fluid [42], [43], local TGF-beta levels in the airways of CF patients may be as high as 2–20 ng/ml, which emphasizes the applicability of the current results to the lower-airway conditions seen in CF patients. Also, we have previously reported that plasma TGF-beta levels are increased during CF pulmonary exacerbations and in pediatric patients with more-severe lung disease based on lung function (forced expiratory volume at 1 second or FEV_1_) segregation and microbiology (with plasma concentrations ranging from 1-7 ng/ml) [41]. While determination of tissue-specific levels of TGF-beta was beyond the scope of this study, the results extend our previous observations and those of other investigators, providing evidence of direct, inverse relationships between chloride-channel activity and TGF-beta exposure in two distinct CF-affected epithelia.

Although TGF-beta has been identified as a genetic modifier of the CF phenotype in three separate cohorts [15], [16], [44], [45], the mechanism of disease modification attributable to TGF-beta remains elusive. TGF-beta is a complex signaling molecule that regulates a number of critical cell functions including growth, senescence, proliferation, and repair [46], [47]. Changes in cellular behavior are governed by activation of TGF-beta receptors, with subsequent triggering of signaling pathways that change gene expression. Activation of the transcription factors pSmad-2/3 leads to their translocation from the cytoplasm to the nucleus, where they regulate the expression of several epithelial and mesenchymal genes. Smad-independent pathways are also activated by TGF-beta receptors, including stimulation of p38 MAPK, Akt, and JNK [48]–[51]. These cell-signaling pathways influence the expression of additional target-gene networks that can result in tissue fibrosis [52]. Our data indicate that TGF-beta activation of pSmad-2/3 contributes to the downregulation of TMEM16A (Figure 8A and C), and activation of phosphor p38 MAPK contributes to the downregulation of CFTR (Figure 8B and D), Complimentary control experiments concluded that the p38 MAPK inhibitor effects were specific for CFTR (with no rescue of TMEM16A expression following TGF-beta treatment - Figure S5B and D). Similar experiments confirmed that the Smad-2/3 inhibitor did not rescue TGF-beta downregulated expression of CFTR in either T84 cells or HAECs (Figure S5A and C).

Comparisons of TGF-beta effects on I_sc_ and protein and transcript levels are summarized in Table 1. CaCC currents and TMEM16A protein levels were reduced 61–67%, whereas TMEM16A transcript levels were only reduced 38–46% in both T84 cells and HAECs. Thus, TMEM16A protein levels and CaCC currents followed parallel sensitivity to TGF-beta that exceeded TMEM16A transcriptional effects. For CFTR, transcript levels were reduced 45% and 71% in T84 cells and HAECs, respectively, whereas CFTR conductance was reduced 94–98% in both cell types. These results therefore support the hypothesis that TGF-beta effects on chloride-channel function include transcriptional and post-transcriptional inhibition. Although part of the discrepancy between chloride-channel expression and function could represent TGF-beta influences on basolateral chloride-entry pathways, the persistent inhibitory effects produced by TGF-beta in the presence of basolateral permeabilization suggest that its effects included apical-membrane transporters (Figure 2A–D). Inhibitors of Smad3 and p38 partially rescued TMEM16A and CFTR protein levels (Figure 8A and C), but failed to consistently rescue CFTR and CaCC function in either cell type (data not shown), suggesting that the total effects of TGF-beta may indeed include additional downregulatory effects on chloride-entry pathways in the basolateral membrane. This broad-based downregulation of ion transport activity by TGF-beta appears logical, since ion transport is a critical function of fully differentiated epithelia, and TGF-beta treatment can lead to a less-differentiated cell phenotype [19]–[21]. The downregulatory effect of TGF-beta on epithelial ion-transport function is further supported by our observation of decreased CaCC, CFTR and amiloride-sensitive currents (a surrogate for ENaC) in HAECs and the disruption of normal ASL-volume regulation (Figure 4). A recent study also supports our finding as TGF-beta disrupts ion and fluid transport in the murine lung by downregulating ENaC in vivo [53]. Further defining these pathways may identify therapeutic targets that enhance chloride-transporter function in conditions of high TGF-beta activity, including optimized rescue of F508del CFTR. Indeed, VX-809 monotherapy has been shown to reduce sweat-chloride levels in a dose-dependent manner in F508del homozygous CF patients, but these effects were not associated with detectable improvements in airway F508del CFTR function (by nasal potential difference measurements), F508del CFTR trafficking (in rectal biopsies), or lung function [28]. These data support the notion that factors within CF-affected tissue compartments could contribute to differential modulator efficacy relative to the sweat gland.

Post-transcriptional regulation of TMEM16A has not been fully described, and post-transcriptional regulation of CFTR is complex, involving numerous binding partners in the endoplasmic reticulum [e.g., Hsp70, Hsc70, and Hdj-1 [54], [55]], the Golgi apparatus and post-Golgi compartment [e.g., CAL (CFTR-associated ligand), TC10, and Snare [56], [57]], and endosomes [58]–[60]. Furthermore, mature CFTR at the plasma membrane can be targeted for internalization and rapid degradation [61]. Therefore, although our data do not identify which of these (or other) steps are responsible for the more-pronounced effects of TGF-beta on chloride-channel function and protein levels relative to transcription, our findings do demonstrate that CFTR may be particularly susceptible to TGF-beta effects in different cell types. TGF-beta also has been shown to downregulate other ion channels in CFTR-affected epithelia [25], which is consistent with the loss of key epithelial functions produced by TGF-beta.

Based on our results, we have established clear and testable hypotheses regarding how local tissue TGF-beta signaling may impact the CF phenotype, potentially modifying CF disease (or other diseases) through effects on CaCC, TMEM16A, and CFTR. For example, increased TGF-beta conditions in the airway of CF patients may be predicted to reduce CaCC-dependent chloride transport, leaving the airway epithelium vulnerable to mucus stasis, infection, and inflammation. In non-CF conditions, elevated tissue levels of TGF-beta may be predicted to reduce CFTR and CaCC activity with potential downstream effects. Although both of these conditions are currently speculative and need to be interpreted in the context of effects of TGF-beta on sodium transport, they provide testable mechanisms to examine how TGF-beta influences the CF phenotype through modulation of ion transport.

## Supporting Information

Figure S1 **Examples of CaCC and CFTR activation and blockade by specific channel inhibitors.** T84 cells in symmetric medium (**A and B**) were pretreated with indomethacin (10 µM apical and basolateral) for 30 min followed by amiloride (‘Amil’, 100 µM apical) for 20 min prior to stimulation. To activate CaCC-dependent transport (**A**), cells were stimulated with ionomycin (‘Iono’ 2 µM apical and basolateral) to increase calcium followed by carbachol (‘Cch’ 100 µM basolateral). CaCC was blocked by tannic acid (500 µM apical).To activate CFTR-dependent transport (**B**), cells were stimulated with forskolin/IBMX (‘Fsk’ 10 µM plus IBMX 100 µM apical and basolateral) to increase cAMP followed by carbachol (‘Cch’ 100 µM basolateral) to activate basolateral potassium channels and increase the electrochemical force for chloride transport (producing a large current spike in the presence of open chloride channels in the apical membrane). Cells were then treated with CFTR_inh172_ (‘Inh172’ 10 µM apical) to block CFTR currents. (**C and D**) HAECs were studied with a chloride secretory gradient. Conditions were similar to those used in T84 cells, except that HAECs were not pretreated with indomethacin, and genistein (‘Gen’ 50 µM apical) replaced Cch to potentiate CFTR.
(TIF)

Figure S2 **Effects of TGF-beta on the calcium and cAMP production on T84 cells and HAECs.** T84 cells or HAECs were treated with TGF-beta (10 ng/ml) or vehicle for 48 h and then stimulated with either forskolin/IBMX (‘F/I’ 10 µM/100 µM), ionomycin (‘Iono’ 2 µM), or ionomycin and carbachol (‘Cch’ 100 µM) for 5 min. Calcium (**A and C**) was measured by ratioed fluorescence of fura-2AM (340/380 nm; Life Technologies, Grand Island, NY) as previously described [62] and cAMP (**B and D**) was measuredby cAMP ELISA kits (Cayman, MI). Calcium and cAMP levels were similar between control and TGF-beta conditions in both T84 cells (**A and B**) and HAECs (**C and D**), *P*>0.05.
(TIF)

Figure S3 **TGF-beta treatment upregulated phosphor p38 and pSmad3 expression in T84 cells and HAECs.** Lysates of T84 cells (**A and B**) or HAECs (**C and D**) were prepared and subjected to PAGE and immunoblot with either anti- pSmad3 or anti- phosphor p38 antibody. For each cell type, the upper gel panels show pSmad3 (**A and C**) or phosphor p38 (**B and D**) detection from three replicate samples (with or without 10 ng/ml TGF-beta exposure). The lower panels are summary densitometry data. **T84 cells:** *P<0.05 for pSmad3; *P<0.001 for phosphor p38. **HAECs:** *P<0.05 for pSmad3; *P<0.001 for phosphor p38.
(TIF)

Figure S4 **Immunofluorescent detection of e-cadherin (green), MUC5AC (red), or acetyl tubulin (purple) in primary polarized HAECs.** HAECs grown on transwell inserts were fixed overnight at 4°C with 4% paraformaldehyde in 0.1 M Phosphate Buffered Saline (PBS). Whole mount immunofluorescence staining was performed on transwell inserts. The samples were permeabilized with 1% Triton X-100 in PBS for 15 min and blocked in PBS containing 5% normal donkey serum for 3 hours at room temperature. The samples were then incubated with primary antibodies [e-Cadherin 1∶100 (Cell Signaling, MA), Acetylated Tubulin 1∶3000 (Sigma, MO) and Muc5AC 1;100 (Abcam, MA)] for 24 hours at 4°C. The samples were then washed three times with PBS, followed by incubation for 2 hours with the respective fluorophore-conjugated secondary antibodies. The samples were washed four times with PBS and counter-stained with DAPI (1 µg/ml). Inserts were mounted on a slide with a No. 1.5 coverslip. Immunofluorescence images were acquired using a Nikon A1Rsi inverted confocal microscope with a 60X WI NA 1.27 objective using a 1.5AU pinhole resulting in a 0.84 µm optical section. Multi-labeled Z-stack images with a 0.28 µm interval between optical sections were acquired sequentially using channel series. Z-intensity correction was used to compensate for reduced signal intensity at increasing Z-depth. Z-stacks were projected in slice view using the Nikon NIS-Elements software. The 3D volume was created in Imaris (Bitplane) using the surpass view and "snapshot" to capture projections. E-cadherin staining was disrupted by TGF-beta treatment (A; B - Z stack) while MUC5AC and acetyl tubulin detection were unaffected.
(TIF)

Figure S5 **No detectable effects of phosphor p38 inhibition on TMEM16A expression, or pSmad3 inhibition on CFTR expression following TGF-beta treatment.** T84 cells (**A and B**) and HAECs (**C and D**) were treated with TGF-beta or TGF-beta plus SB203580 (p38 inhibitor - **A and C**) or TGF-beta or TGF-beta plus SIS3 (pSmad3 inhibitor - **B and D**) for 48 h prior to lysis and immunoblot for either TMEM16A (**A and C**) or CFTR (**B and D**). TGF-beta reduced TMEM16A and CFTR expression in both cell types in the presence or absence of tested inhibitors*P<0.01 for TMEM16A; *P<0.003 for CFTR compared with untreated conditions). SIS3-treated T84 cells and HAECs had no rescue effects on CFTR expression.
(TIF)
