# Supplementary figures and images for: TGF-Beta Downregulation of Distinct Chloride Channels in Cystic Fibrosis-Affected Epithelia
Source: PLoS One. 2014 Sep 30;9(9):e106842. doi: 10.1371/journal.pone.0106842 (PMC4182049; doi:10.1371/journal.pone.0106842)

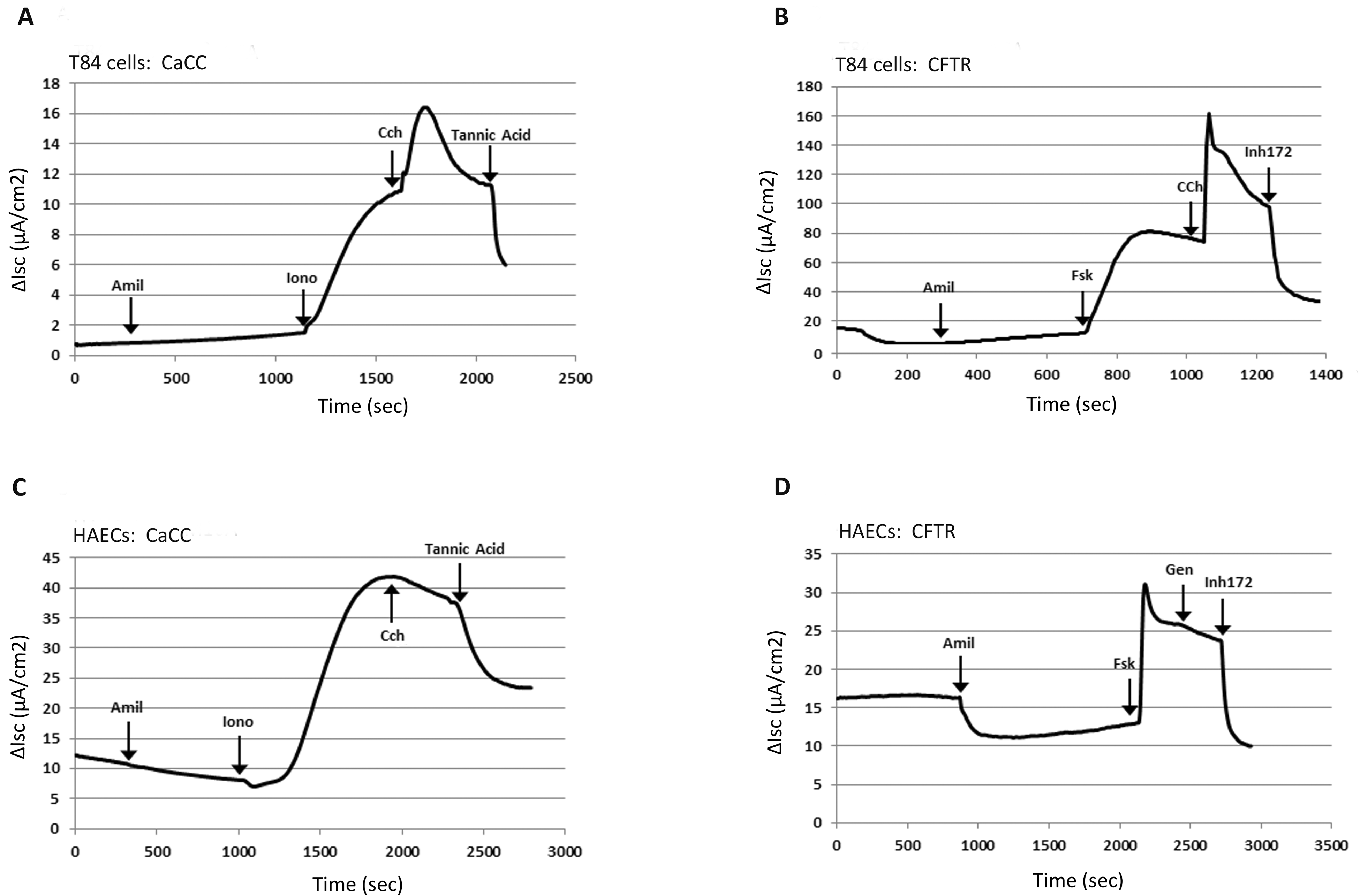

Supplement: Figure S1 — Examples of CaCC and CFTR activation and blockade by specific channel inhibitors. T84 cells in symmetric medium (A and B) were pretreated with indomethacin (10 µM apical and basolateral) for 30 min followed by amiloride (‘Amil’, 100 µM apical) for 20 min prior to stimulation. To activate CaCC-dependent transport (A), cells were stimulated with ionomycin (‘Iono’ 2 µM apical and basolateral) to increase calcium followed by carbachol (‘Cch’ 100 µM basolateral). CaCC was blocked by tannic acid (500 µM apical).To activate CFTR-dependent transport (B), cells were stimulated with forskolin/IBMX (‘Fsk’ 10 µM plus IBMX 100 µM apical and basolateral) to increase cAMP followed by carbachol (‘Cch’ 100 µM basolateral) to activate basolateral potassium channels and increase the electrochemical force for chloride transport (producing a large current spike in the presence of open chloride channels in the apical membrane). Cells were then treated with CFTRinh172 (‘Inh172’ 10 µM apical) to block CFTR currents. (C and D) HAECs were studied with a chloride secretory gradient. Conditions were similar to those used in T84 cells, except that HAECs were not pretreated with indomethacin, and genistein (‘Gen’ 50 µM apical) replaced Cch to potentiate CFTR. (TIF) [file pone.0106842.s001.tif]

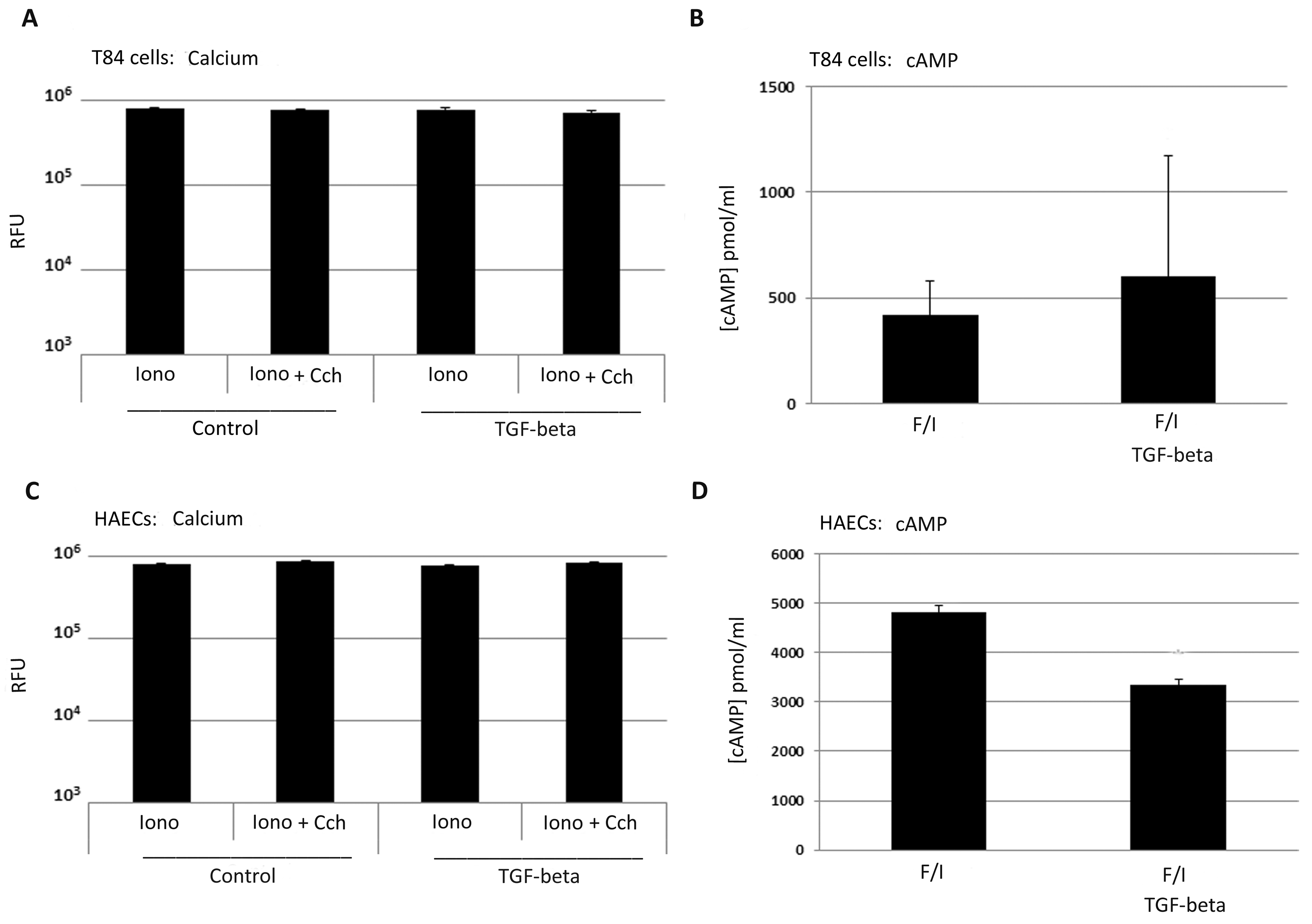

Supplement: Figure S2 — Effects of TGF-beta on the calcium and cAMP production on T84 cells and HAECs. T84 cells or HAECs were treated with TGF-beta (10 ng/ml) or vehicle for 48 h and then stimulated with either forskolin/IBMX (‘F/I’ 10 µM/100 µM), ionomycin (‘Iono’ 2 µM), or ionomycin and carbachol (‘Cch’ 100 µM) for 5 min. Calcium (A and C) was measured by ratioed fluorescence of fura-2AM (340/380 nm; Life Technologies, Grand Island, NY) as previously described [62] and cAMP (B and D) was measuredby cAMP ELISA kits (Cayman, MI). Calcium and cAMP levels were similar between control and TGF-beta conditions in both T84 cells (A and B) and HAECs (C and D), P>0.05. (TIF) [file pone.0106842.s002.tif]

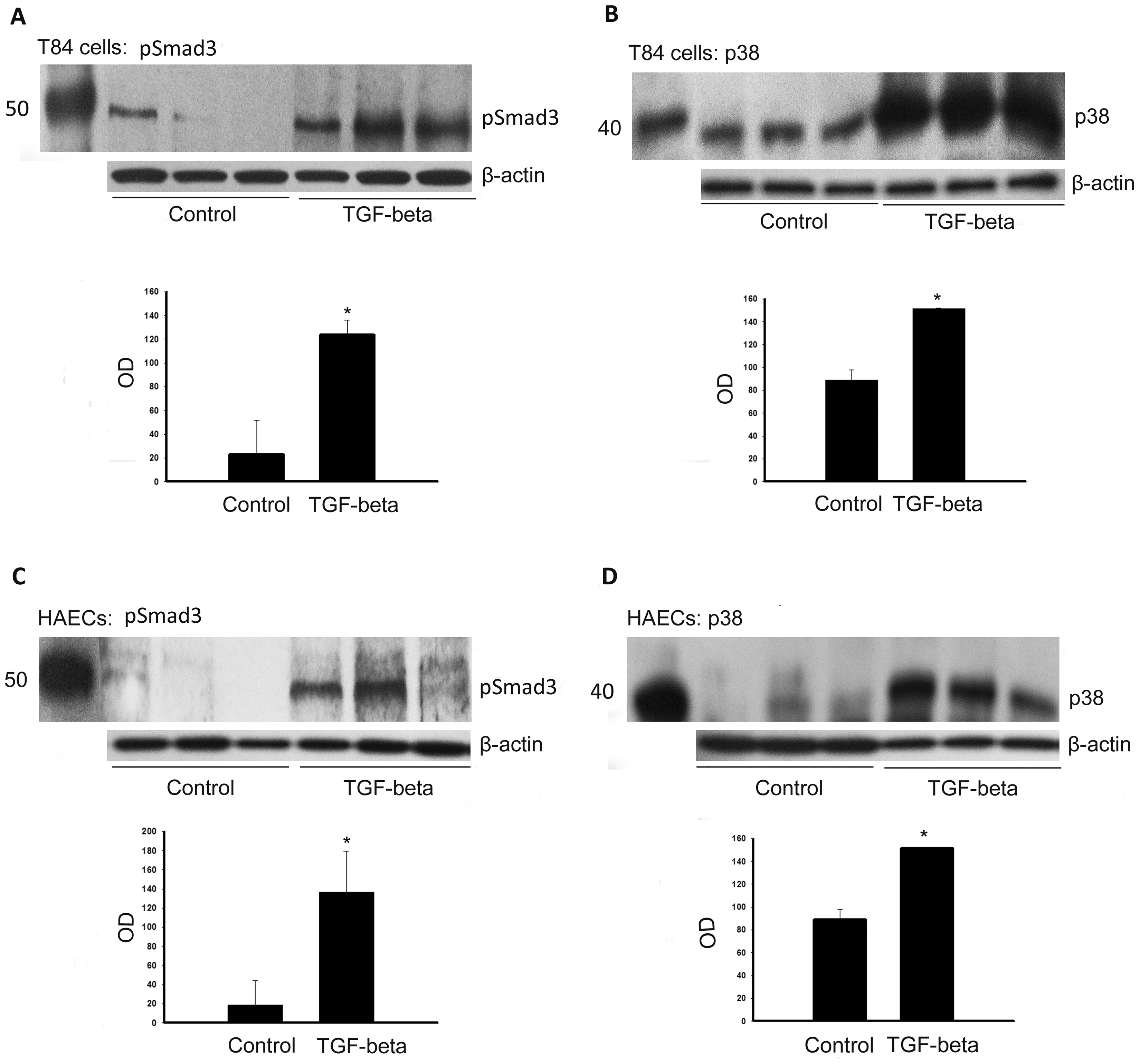

Supplement: Figure S3 — TGF-beta treatment upregulated phosphor p38 and pSmad3 expression in T84 cells and HAECs. Lysates of T84 cells (A and B) or HAECs (C and D) were prepared and subjected to PAGE and immunoblot with either anti- pSmad3 or anti- phosphor p38 antibody. For each cell type, the upper gel panels show pSmad3 (A and C) or phosphor p38 (B and D) detection from three replicate samples (with or without 10 ng/ml TGF-beta exposure). The lower panels are summary densitometry data. T84 cells: *P<0.05 for pSmad3; *P<0.001 for phosphor p38. HAECs: *P<0.05 for pSmad3; *P<0.001 for phosphor p38. (TIF) [file pone.0106842.s003.tif]

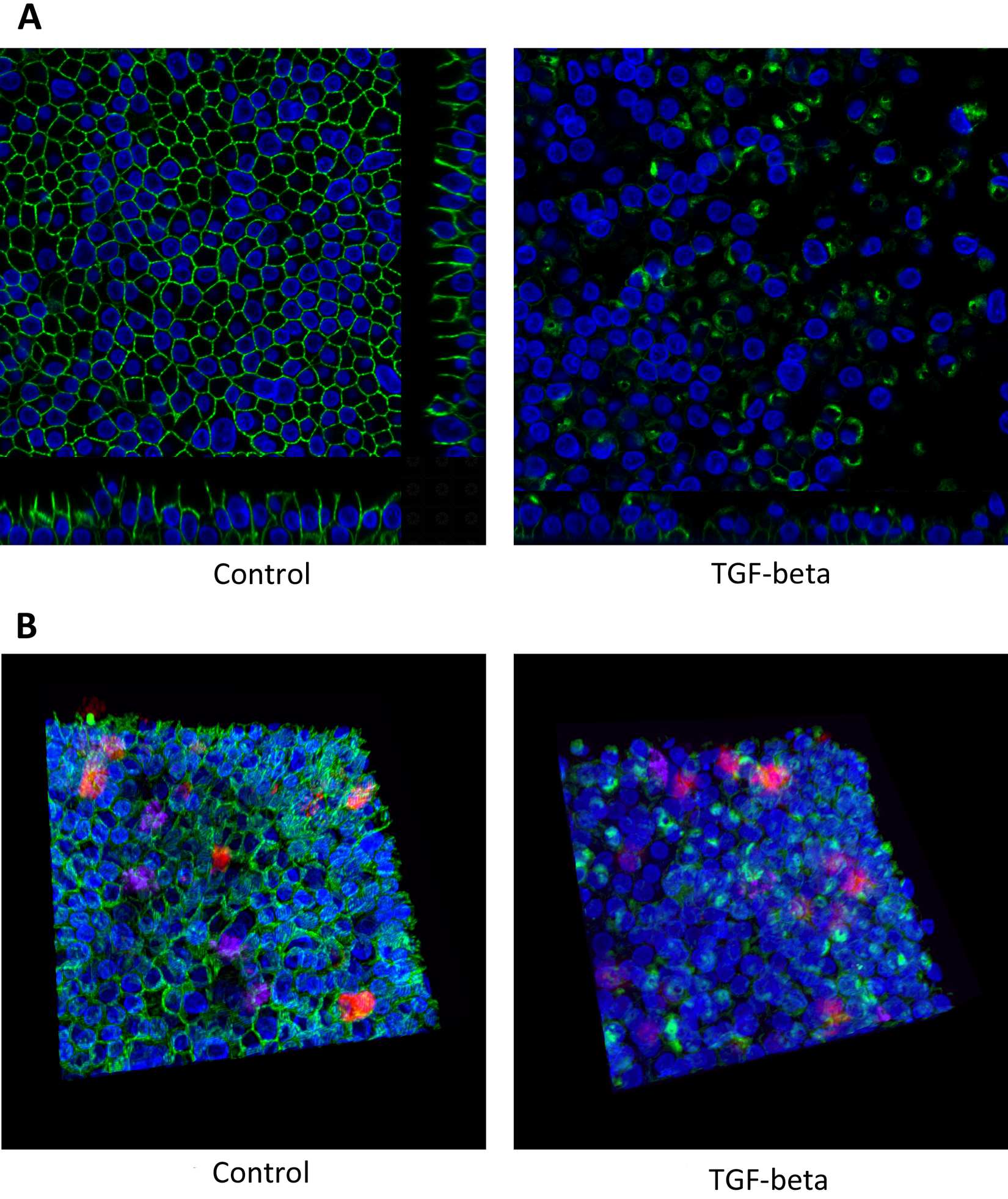

Supplement: Figure S4 — Immunofluorescent detection of e-cadherin (green), MUC5AC (red), or acetyl tubulin (purple) in primary polarized HAECs. HAECs grown on transwell inserts were fixed overnight at 4°C with 4% paraformaldehyde in 0.1 M Phosphate Buffered Saline (PBS). Whole mount immunofluorescence staining was performed on transwell inserts. The samples were permeabilized with 1% Triton X-100 in PBS for 15 min and blocked in PBS containing 5% normal donkey serum for 3 hours at room temperature. The samples were then incubated with primary antibodies [e-Cadherin 1∶100 (Cell Signaling, MA), Acetylated Tubulin 1∶3000 (Sigma, MO) and Muc5AC 1;100 (Abcam, MA)] for 24 hours at 4°C. The samples were then washed three times with PBS, followed by incubation for 2 hours with the respective fluorophore-conjugated secondary antibodies. The samples were washed four times with PBS and counter-stained with DAPI (1 µg/ml). Inserts were mounted on a slide with a No. 1.5 coverslip. Immunofluorescence images were acquired using a Nikon A1Rsi inverted confocal microscope with a 60X WI NA 1.27 objective using a 1.5AU pinhole resulting in a 0.84 µm optical section. Multi-labeled Z-stack images with a 0.28 µm interval between optical sections were acquired sequentially using channel series. Z-intensity correction was used to compensate for reduced signal intensity at increasing Z-depth. Z-stacks were projected in slice view using the Nikon NIS-Elements software. The 3D volume was created in Imaris (Bitplane) using the surpass view and "snapshot" to capture projections. E-cadherin staining was disrupted by TGF-beta treatment (A; B - Z stack) while MUC5AC and acetyl tubulin detection were unaffected. (TIF) [file pone.0106842.s004.tif]

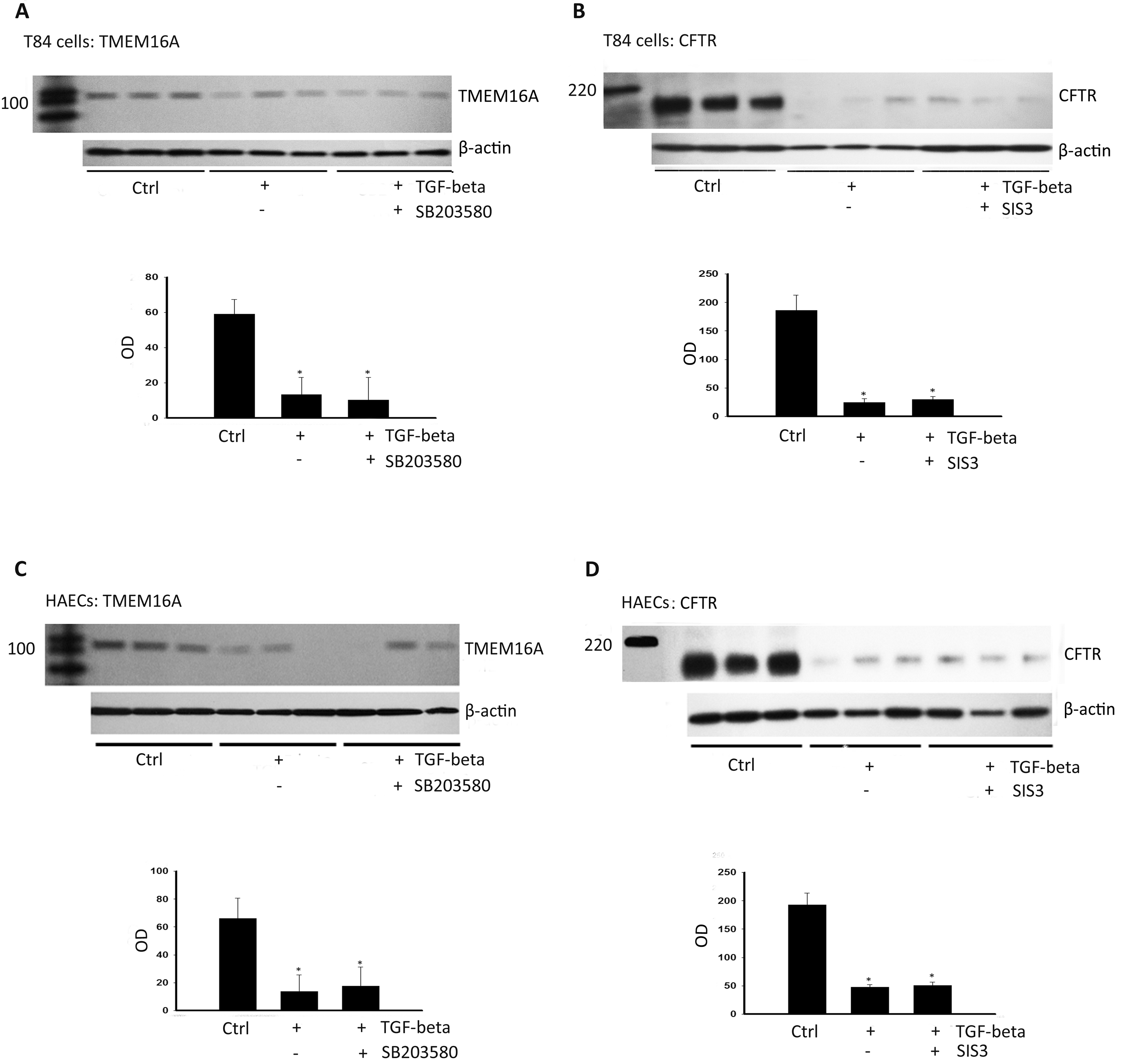

Supplement: Figure S5 — No detectable effects of phosphor p38 inhibition on TMEM16A expression, or pSmad3 inhibition on CFTR expression following TGF-beta treatment. T84 cells (A and B) and HAECs (C and D) were treated with TGF-beta or TGF-beta plus SB203580 (p38 inhibitor - A and C) or TGF-beta or TGF-beta plus SIS3 (pSmad3 inhibitor - B and D) for 48 h prior to lysis and immunoblot for either TMEM16A (A and C) or CFTR (B and D). TGF-beta reduced TMEM16A and CFTR expression in both cell types in the presence or absence of tested inhibitors*P<0.01 for TMEM16A; *P<0.003 for CFTR compared with untreated conditions). SIS3-treated T84 cells and HAECs had no rescue effects on CFTR expression. (TIF) [file pone.0106842.s005.tif]
